# Potential Occupational Risks Associated with Pulmonary Toxicity of Carbon Nanotubes

**DOI:** 10.4172/2329-6879.1000165

**Published:** 2014-06-13

**Authors:** Amruta Manke, Sudjit Luanpitpong, Yon Rojanasakul

**Affiliations:** 1 Department of Pharmaceutical Sciences, West Virginia University, Morgantown, USA; 2 Mary Babb Randolph Cancer Center, West Virginia University, Morgantown, WV 26506, USA

**Keywords:** Carbon nanotube, Pulmonary toxicity, Worker exposure, Fibrosis, Occupational hazard

## Abstract

Given their remarkable properties, carbon nanotubes (CNTs) have made their way through various industrial and medicinal applications and the overall production of CNTs is expected to grow rapidly in the next few years, thus requiring an additional recruitment of workers. However, their unique applications and desirable properties are fraught with concerns regarding occupational exposure. The concern about worker exposure to CNTs arises from the results of recent animal studies. Short-term and sub-chronic exposure studies in rodents have shown consistent adverse health effects such as pulmonary inflammation, granulomas, fibrosis, genotoxicity and mesothelioma after inhalation or instillation of several types of CNTs. Furthermore, physicochemical properties of CNTs such as dispersion, functionalization and particle size can significantly affect their pulmonary toxicity. Risk estimates from animal studies necessitate implementation of protective measures to limit worker exposure to CNTs. Information on workplace exposure is very limited, however, studies have reported that CNTs can be aerosolized and attain respirable airborne levels during synthesis and processing activities in the workplace. Quantitative risk assessments from sub-chronic animal studies recommend the health-based need to reduce exposures below the recommended exposure limit of 1 µg/m^3^. Practice of prevention measures including the use of engineering controls, personal protective equipment, health surveillance program, safe handling and use, as well as worker training can significantly minimize worker exposure and improve worker health and safety.

## Introduction

Since their discovery, carbon nanotubes (CNTs) have been considered as ideal candidate for applications in industries such as electronics, energy, pharmaceuticals, cosmetics, agriculture and medical diagnostics due to their unique physicochemical properties including high tensile strength and conductivity [1–3]. The CNT industry has been projected to grow tremendously over the next decade with global capacity in 2013 estimated at 2,000 tons/year for multi-walled CNT and 6 tons/year for single-walled CNT [4,5]. The National Science Foundation projects that by 2020, the CNT industry will employ approximately 6 million workers, 2 million of whom are expected to be in the United States [6]. Currently, a large number of occupational personnel are known to be involved in the handling of CNTs at 61 companies in the U.S. with a projected annual growth of about 22% [7,8]. The growing production and use of CNTs will result in a dramatic increase in occupational and public exposure to engineered nanomaterials. Since workers are the most likely individuals to be exposed to CNTs given their involvement in handling and producing nanomaterials, occupational safety and health is of utmost importance while dealing with these materials with new risks and uncertainties. Understanding, predicting, and managing the potential health risks associated with CNT exposure in the workplaces where they are being fabricated and incorporated into products is one of the key steps towards human exposure risk assessment [9,10].

As per British Standards Institute Report (2007), CNTs are high aspect ratio nanomaterial’s (HARNs) having at least one of their dimensions, i.e. the diameter less than 100 nm whereas their length may be in the micrometer range. They are classified into two main types known as single-walled CNT (SWCNT) and multi-walled CNT (MWCNT) [11]. A growing body of literature indicates potential health hazard to workers from CNT exposure. Owing to their bio persistence, surface reactivity and asbestos-like properties, CNTs are believed to induce biologically harmful effects via their ability to translocate within the alveolar regions and the deeper pleura of the lung [12,13]. Inhalation being the major route of exposure for CNTs, accumulating evidence indicates the pulmonary pathologic responses such as inflammation, oxidative stress, granulomas, genotoxicity, pleural and interstitial fibrosis following CNT exposure [14–16]. Data from existing animal studies as well as reported workplace airborne CNT levels strongly suggest the need to minimize worker exposure and protect worker health [17]. Additional research is required to determine how airborne CNTs in the workplace may compare in size and structure to the aerosolized CNTs generated in the animal and *in vitro* studies [9]. In this article, we will describe and summarize the adverse respiratory health effects documented in animal models following CNT exposure, pathological effects of CNTs influenced by their physicochemical properties and discuss the potential sources for workplace CNT exposure and the recommended limits for CNT exposure followed by strategies for accurate measurement and control of CNT exposure. For our article, we utilized the technique of narrative review to identify and condense the literature in the area of pulmonary toxicity of carbon nanotubes. We identified various research studies, previously conducted synthesized documents using electronic databases such as PubMed, Ebscohost, Web of Science and Medline. After pooling the studies in a databank, authors identified relevant studies by carefully screening the abstracts. Based on all the relevant studies, we formulated important themes that provide comprehensive knowledge about the area of pulmonary toxicity of CNTs. The information from each article was then carefully condensed within these themes.

## Evidence for adverse pulmonary outcomes following CNT exposure

Currently, there are no published reports of the adverse health effects in workers handling CNTs. However, given the likelihood of developing robust pulmonary responses after inhalation of particles and fibers, it is rational to assume that at an equivalent lung burden to CNTs, workers may also be susceptible to developing these adverse lung effects. Nevertheless, mounting evidence from animal studies raises serious health concerns for occupational hazards associated with CNT exposure. Due to the lack of safety guidelines and suitable biomarkers for CNT workplace exposure, occupational risk estimates are extrapolated from existing rodent models [9,14].

## Pulmonary toxicity of CNTs *in vivo*

### Fibrosis

Over the past few years, there has been a considerable growth in the literature base documenting dose and time-dependent biological effects of SWCNT and MWCNT exposure. Some early reports provided evidence for intrinsic toxicity of CNTs and potential exposure to respirable CNT particulate matters in workers [12,11]. Pharyngeal aspiration of SWCNT in mice at the dose of 10–40 µg/mouse induced acute inflammation, early onset of granulomas, alveolar wall thickening, and progressive fibrosis [11]. Subsequent studies determined the influence of the route of administration and dispersion status on the end toxic response. For instance, a short-term SWCNT inhalation exposure was more effective than pharyngeal aspiration in causing lung toxicity in mice as evidenced by a 4-fold greater inflammation and fibrosis than aspiration of the same mass lung burden owing to aerosolized particle size during inhalation [15,16]. Poorly dispersed SWCNT in suspension was found to be restricted to the proximal alveolar regions resulting in granulomatous lesions, whereas well-dispersed SWCNT deposited deeper into the interstitial and pleural areas of the lung causing parenchymal granulomas and interstitial fibrosis [18,19]. Likewise, the biopersistence of MWCNT has been illustrated by a number of *in vivo* studies suggesting similar spectrum of dose- and time-dependent pulmonary fibrogenic responses [13,20,21]. Contrary to SWCNT, MWCNT was shown to induce a significant increase in fibrosis after pulmonary aspiration compared to inhalation [22]. Acute pulmonary exposure to inhaled MWCNT induced inflammation, fibrosis, and rare pleural penetration indicating that MWCNT can reach the pleura after inhalation [23]. A recent long-term inhalation study demonstrated that MWCNT induced a fibrotic response that persisted up to 336 days post-exposure and exhibited particle size-dependent retention in the lungs [24]. Furthermore, inhaled MWCNT were found to translocate to the parietal pleura, the respiratory musculature, liver, kidney, heart and brain where they accumulate with time following exposure [25].

Despite similar qualitative fibrogenic responses, both MWCNT and SWCNT differ significantly in how they are distributed within the lungs. SWCNT is more likely to interact with the lung owing to its greater fiber count per mass than MWCNT [22]. Moreover, MWCNT is known to be recognized and phagocytosed by alveolar macrophages [20,26], whereas SWCNT evades macrophages which facilitates its entry into the alveolar interstitium [22]. However, both forms of CNT induce damaging lung responses *in vivo* at doses physiologically relevant to potential worker exposures. Pulmonary exposure to CNTs has illustrated systemic responses such as increased inflammatory mediators in the blood, diminished ability of coronary arterioles to respond to dilators, oxidative stress in aortic tissue and increased plaque formation in an atherosclerotic mouse model [27–29]. Additional research is needed to understand the mechanisms underlying these pulmonary and systemic responses to CNTs.

Besides the fibrogenic damage, long-term CNT exposure has been shown to promote malignant transformation and induction of tumorigenesis, initiation of lung adenocarcinoma and tumor like morphology *in vivo* at doses which approximate potential human occupational exposures [30,31].

### Pulmonary function

CNTs can significantly hamper pulmonary function as demonstrated by an increase in expiratory time [15], reduced bacterial clearance activity [16], and decreased lung compliance [32]. Both SWCNT and MWCNT exacerbate ovalbumin-induced allergic airway inflammation *in vivo* [33–35]. Collectively, these studies imply that individuals with pre-existing respiratory conditions such as allergic asthma and bronchitis are more likely to be susceptible to CNT exposure [36].

### Pleural disease

The structural similarity between asbestos and CNTs has raised a concern about the potential damaging effect of CNTs on pleural mesothelium. Studies have demonstrated CNTs to reach the pleural space [26], migrate from subpleural to intrapleural tissue [37], induce mesothelial cell proliferation and mesothelioma formation [38,39], and cause inflammation and pleural fibrosis [22]. MWCNT injected into the peritoneal cavity of mice or rats generated fiber length-dependent inflammation/genetic damage and mesothelioma [40]. These findings are important in understanding whether CNTs have the potential to cause asbestos-like pleural lesions and whether workers are at risk of developing mesothelioma after chronic CNT exposure.

## Pulmonary toxicity *in vitro*

### Oxidative stress

Numerous studies have indicated CNT-induced ROS generation in multiple cell lines [20,41–43] and activation of ROS-associated intracellular signaling pathways including mitogen activated protein kinase (MAPK), Akt, activator protein-1 (AP-1), and nuclear factor kappa-light-chain-enhancer of activated B cells (NF-κB) in mesothelial cells in a dose-dependent manner [44]. These findings indicate that CNT-induced oxidative stress may serve as an important intermediate endpoint for assessment of CNT-induced pulmonary toxicity.

### Epithelial barrier permeability

Since epithelial cells are the first line of defense against inhaled foreign particles, disturbance in respiratory barrier function is critical to CNT-induced toxicity. Studies have shown the translocation of engineered nanoparticles across the alveolar epithelial monolayers *in vitro* which corresponded to the delivery of particles in suspension to cells, thereby highlighting the need for accurate dosimetry when investigating biological interactions with CNTs [45]. CNTs have been shown to alter the paracellular permeability of airway epithelial cells and inhibit mucociliary clearance mechanisms by interfering with the formation of tight junctions [46–48]. Such alteration would likely impair the protective barrier function of lung epithelium.

### Genotoxicity

Since CNTs have been shown to possess asbestos-like pathogenicity, it is necessary to characterize their genotoxic potential. Both MWCNT and SWCNT have been shown to exert genotoxic effects in a number of *in vitro* settings as evidenced by DNA strand breakage, DNA base oxidation, chromosomal aberrations and gene mutations [49–55]. Intrinsic ROS production, CNT-induced inflammation and oxidative stress are some of the proposed mechanisms for CNT-driven genotoxicity [2,56,57]. More recent studies have revealed the potential toxicity associated with chronic exposure of CNTs which results in a malignant and neoplastic phenotype and tumorigenesis as well a novel feature of stem-like induction in human lung cells [58,59]

## Physicochemical characteristics of CNTs

The physical-chemical characteristics of CNTs such as size, chemical composition and surface charge can often be modified to accommodate their intended commercial use and to improve their biocompatibility, thereby conferring them new functions which cannot otherwise be acquired by pristine CNTs [2,3,9]. However, these changes often translate into altered biological reactivity of CNTs, which is poorly understood at present [2,56]. Additional information is required to characterize the relationship between physicochemical properties of CNTs and their bioactivity in order to predict occupational health hazard and safer design of CNTs. The influence of physicochemical parameters of CNTs on their biological activities is further described in details in Tables 1–3.

## Workplace exposure to CNTs

Human exposure to manufactured nanomaterials is most likely to be observed in workers than the general population [60]. CNT exposure may occur at a multitude of stages such as laboratory research, nanomaterial synthesis, downstream use and disposal/recycle of CNTs [61]. The duration of exposure to airborne CNT particles and the release, dispersion and transformation of CNTs dictate the occupational risk [62]. However, the extent to which workers are exposed remains to be fully characterized since information on workplace exposure is limited. The initial report on laboratory and field measurements of CNT exposure demonstrated significant aerosolization of CNTs. Particle measurements during the study indicated CNT mass concentrations in the 0.7 to 53 µg/m^3^ range, in the form of agglomerates with sizes typically larger than 1 µm. Additionally, these agglomerates were retained in the workspace atmosphere for a long period of time given their low bulk density, and the majority of these particles were respirable [61,63]. Subsequent studies demonstrated CNT aerosolization resulting in appreciable airborne CNT levels as high as 430 µg/m^3^ during weighing, transfer, cutting and sonication in a laboratory setting [64–67]. These findings demand implementation of robust measures to protect workers health and need for in-house controls to curtail worker exposure. Employing airborne particle concentrations as a surrogate marker for measuring the potential release of CNTs (e.g. local exhaust ventilation, wet cutting of composites, fume hood/enclosures) effectively reduced the worker exposure demonstrating that conventional industrial measures can effectively lessen airborne levels [65,66,68]. Since CNTs often occur as micrometer-sized aggregates, newer studies have employed a specific marker for CNT exposure, i.e. NMAM 5040, which measures the specific mass of elemental carbon and provides a more refined mass [69]. In order to identify and measure occupational hazards associated with CNT exposure, the National Institute for Occupational Safety and Health (NIOSH) has proposed a recommended exposure limit (REL) of 1 µg/m^3^ for CNT exposure based on quantitative risk assessment and estimates from sub-chronic and short-term animal studies with dose-response data of early stage fibrotic and inflammatory lung responses to CNT exposure [9]. The NIOSH report recommends that workers may have >10% higher risk of developing early-stage pulmonary fibrosis if exposed to the REL over working lifetime of about 45 years. This respirable mass-based REL is a workplace barometer to identify job tasks with potential exposures to CNTs and guarantee that appropriate measures are taken to limit worker exposure [9,70]. Thus efforts should be made to reduce airborne concentrations of CNTs as low as possible below the REL.

Given the uncertainties regarding risk assessment of occupational exposures to CNT, many studies have recommended using occupational exposure limits (OELs) which are defined using benchmark particles which in turn are evaluated using standard criteria [9,71,72]. The very initial studies reported a benchmark OEL of 0.01 fiber/cm^3^ for CNT based on an existing OEL for asbestos [72–74]. Currently, the suggested OELs for CNT range from 1–50 µg/m^3^ as indicated by subsequent studies [75–77]. Another risk-based approach suggested comparing the potency and mode of action to benchmark materials for CNT exposure assessment [72,78].

Given the current workplace CNT concentrations, available animal data indicate that over a working lifetime, workers may be vulnerable to CNT-prone adverse pulmonary effects. Due to the limited information about human exposure and risks, it is recommended that prevention strategies and control measures to minimize exposures be adopted [9]. A general occupational medical surveillance program is recommended for workers and worksites. Workers should be trained to anticipate, identify, and track potentially hazardous nanomaterials in the workplace [10]. For safe handling and application, periodic evaluation of the potential health risks associated with CNT exposure is essential. Appropriate use of engineering controls such as use of fume hoods and exhaust ventilation and personal protective equipment (e.g. respirators) should be mandated for worker safety [9,17,61].

Precise analytic measurement methods are required to provide workplace exposure data and establish exposure standards [79]. Such exposure data would guide long-term inhalation studies to determine the time course and dose response for possible development of fibrosis, cancer and mesothelioma. Identifying key biomarkers, mechanistic endpoints would be useful in worker exposure surveillance [61]. More animal studies are required to understand the influence of physicochemical properties on the bioactivity of CNTs for safer design and use of CNT technology.

## Concluding remarks

This review article addresses the impending issue of adverse health impacts on workers with higher likelihood of being exposed to potential hazards of nanotechnology. The fibrogenic and genotoxic effects of CNTs raise important health concerns for workers and consumers. There is significant correlation between the pulmonary responses *in vivo* and *in vitro* and the human health risks associated with CNT exposure. Understanding the effect of CNT characteristics on their biological reactivity will contribute towards safer industrial and consumer applications of the nanomaterial. Additional information regarding the mechanisms and diagnostic markers relevant to CNT-induced responses would facilitate risk assessment and predictive toxicity testing. The key is to prevent and minimize inadvertent exposure today via the use of appropriate controls at workplaces, safety equipments and worker training.

## Figures and Tables

**Table 1:** Effect of surface functionalization on CNT-induced pulmonary toxicity *in vitro* and *in vivo*

Type of CNT	System	Effect	Study
SWCNT, control and acid functionalized (AF-SWCNT)	LA4 mouse lung epithelial cells and *in vivo* in CD1 mice	AF-SWCNT more cytotoxic than SWCNT *in vitro*; exerted stronger inflammatory response *in vivo* than control SWCNT	[80]
MWCNT, functionalized and non-functionalized	*In vivo* bone marrow cells of Swiss-Webster mice	Functionalized MWCNT induced greater clastogenic/genotoxic effects than non-functionalized MWCNT	[81]
SWCNT, SWCNT-phenyl-SO3H, SWCNT-phenyl-SO3Na, and SWCNT-phenyl-(COOH)2	Human dermal fibroblasts	Cytotoxicity dependent on the degree of sidewall functionalization	[82]
MWCNT, pristine and carboxylated	*In vivo* mice	The degree of functionalization was inversely proportional to hepatic toxicity	[83]
MWCNT, pristine and functionalized (MW-COOH and MW-NH_2_)	A549 pneumocytes, *in vivo*	pulmonary toxicity, inflammatory response, irrespective of nanotube functionalization	[84]
As-prepared (AP), COOH, PEG, NH_2_, NH_2_, and PEI-MWCNT	BEAS-2B and THP-1 cells, *in vivo*	Chronic lung inflammation, fibrosis, and collagen deposition: PEI-MWCNT induced the strongest effects, while NH_2_ and sw-NH_2_-MWCNT exerted similar effects, and COOH and PEG-MWCNT induced weaker effects than AP-MWCNT *in vitro* and *in vivo*	[85]
MWCNT, CNF, carbon nanoparticles	Human lung tumor cells	Functionalized carbon nanoparticles most toxic compared to MWCNT and CNF	[86]
SWCNT, purified and 6-amino-hexanoic acid-derivatized (AHA-SWCNT)	Human epidermal keratinocytes	Functionalization induced mild cytotoxic responses and maintained cell viability	[87]

**Table 2:** Effect of size and surface area on CNT-induced lung toxicity *in vitro* and *in vivo*

Type of CNT	System	Effect	Study
Purified MWCNT, short (220 nm) and long (825 nm)	Human acute monocytic leukemia THP-1 cell line	Long CNT induced more inflammation	[88]
SWCNT, long (0.5–100 µm) and short (0.5–2 µm), MWCNT, long (5–9 µm) and short (0.5–2 µm)	Human epithelial Calu-3	Long MWCNT and SWCNT caused significant disruption of barrier function	[47]
MWCNT long (13 µm) and (56 µm), tangled (1–5 µm) and (5–20 µm)	*In vivo*	Length-dependent inflammation and granuloma formation	[37]
MWCNT, short (1–10 µm), long tangled (10–50 µm), long needle-like (>50 µm), asbestos (4.6 µm) and carbon black	Primary human macrophages	Enhanced activation of NRLP3 inflammasome and secretion of IL-1β, IL-1α by long MWCNT	[89]
MWCNT, Long, short, tangled, Nickel nanowires, long and short	*In vivo*	Length-dependent retention of CNTs into lung pleura resulting in sustained inflammation and progressive fibrosis	[90]
MWCNT, dispersed thin (50 nm), aggregative (2–20 nm), thick (150 nm)	Human peritoneal mesothelial cells	Thin MWCNT more inflammatory and carcinogenic	[40]
Purified MWCNT, thick (70 nm) and thin (9.4 nm)	Murine alveolar macrophages and in vivo in rats	Thin MWCNT more toxic *in vitro* and *in vivo*	[91]
SWCNT (138 m^2^/g), carbon nanofibers, CNF (21 m^2^/g), asbestos (8 m^2^/g)	*In vivo* C57BL/6 mice	SWCNT with high surface area induced more oxidative stress, inflammation, lung damage and fibrosis than CNF and asbestos	[19]
SWCNT, MWCNT, active carbon, carbon black and carbon graphite	Human fibroblast cells	SWCNT with small surface area more toxic than large particles	[92]
MWCNT, CNF, carbon nanoparticles	Human lung tumor cells	Size and aspect ratio-dependent cytotoxicity of MWCNT	[86]
MWCNT, short and long	Murine macrophages	Short>long MWCNT in pro-inflammatory cytokine secretion and oxidative stress	[93]
MWCNT, (NM400 and NM402) Crocidolite	Human fibroblast cells, *in vivo* C57BL/6 mice	Long MWCNT induced more cell proliferation *in-vitro* and fibrosis *in vivo*	[94]
SWCNT, long SWCNT fibers (~13 µm) Short SWCNT fibers (~1–2 µm)	Human lung fibroblasts, *in vivo* C57BL/6 mice	Length-dependent ROS generation, TGF-β and collagen I expression	[95]

**Table 3:** 

a: Effect of presence of metal impurities on CNT-induced pulmonary toxicity *in vitro* and *in vivo*			
Type of CNT	System	Effect	Study
30 wt% iron-rich SWCNT	Human keratinocytes	Loss of cell viability and oxidative stress were due to the catalytic activity of SWCNT-associated iron content	[96]
99%, acid-treated 97%, and (97% purity surface oxidation 8%) MWCNT	Human neuroblastoma cells	Loss of cell viability with reduction in CNT purity	[97]
26 wt% iron-rich SWCNT	Murine RAW 264.7 macrophages	Loss of intracellular thiols and lipid hydroperoxide accumulation in macrophages	[98]

b: Effect of dispersion status on CNT-induced lung toxicity *in vitro* and *in vivo*			
Type of CNT	System	Effect	Study
SWCNT, poor and well dispersed	*In vivo* C57BL/6 mice	Poorly dispersed SWCNT-proximal alveolar regions resulting in granulomatous lesions; well-dispersed CNT-alveolar interstitial and pleural areas causing parenchymal granulomas and interstitial fibrosis	[18]
SWCNT, Survanta dispersed (SD-SWCNT) and non-dispersed (ND-SWCNT)	Human lung epithelial BEAS-2B cells	SD-SWCNT more fibrogenic than ND-SWCNT both *in vitro* and *in vivo*	[99]
